# SwissMTB: establishing comprehensive molecular cancer diagnostics in Swiss clinics

**DOI:** 10.1186/s12911-018-0680-0

**Published:** 2018-10-29

**Authors:** Franziska Singer, Anja Irmisch, Nora C. Toussaint, Linda Grob, Jochen Singer, Thomas Thurnherr, Niko Beerenwinkel, Mitchell P. Levesque, Reinhard Dummer, Luca Quagliata, Sacha I. Rothschild, Andreas Wicki, Christian Beisel, Daniel J. Stekhoven

**Affiliations:** 1 NEXUS Personalized Health Technologies, ETH Zurich, Otto-Stern-Weg 7, 8093 Zurich, Switzerland; 2 SIB Swiss Institute of Bioinformatics, 4058 Basel, Switzerland; 3 Department of Dermatology, University Hospital Zurich, 8091 Zurich, Switzerland; 4 Department of Biosystems Science and Engineering, ETH Zurich, 4058 Basel, Switzerland; 5 Division of Oncology, Department of Biomedicine, University Hospital Basel, Petersgraben 4, 4031 Basel, Switzerland; 6 Department of Pathology, University Hospital Basel, Schönbeinstrasse 40, 4056 Basel, Switzerland

**Keywords:** Molecular diagnostics, NGS, Personalized medicine, Cancer diagnostics, Molecular tumor board

## Abstract

**Background:**

Molecular precision oncology is an emerging practice to improve cancer therapy by decreasing the risk of choosing treatments that lack efficacy or cause adverse events. However, the challenges of integrating molecular profiling into routine clinical care are manifold. From a computational perspective these include the importance of a short analysis turnaround time, the interpretation of complex drug-gene and gene-gene interactions, and the necessity of standardized high-quality workflows. In addition, difficulties faced when integrating molecular diagnostics into clinical practice are ethical concerns, legal requirements, and limited availability of treatment options beyond standard of care as well as the overall lack of awareness of their existence.

**Methods:**

To the best of our knowledge, we are the first group in Switzerland that established a workflow for personalized diagnostics based on comprehensive high-throughput sequencing of tumors at the clinic. Our workflow, named SwissMTB (Swiss Molecular Tumor Board), links genetic tumor alterations and gene expression to therapeutic options and clinical trial opportunities. The resulting treatment recommendations are summarized in a clinical report and discussed in a molecular tumor board at the clinic to support therapy decisions.

**Results:**

Here we present results from an observational pilot study including 22 late-stage cancer patients. In this study we were able to identify actionable variants and corresponding therapies for 19 patients. Half of the patients were analyzed retrospectively. In two patients we identified resistance-associated variants explaining lack of therapy response. For five out of eleven patients analyzed before treatment the SwissMTB diagnostic influenced treatment decision.

**Conclusions:**

SwissMTB enables the analysis and clinical interpretation of large numbers of potentially actionable molecular targets. Thus, our workflow paves the way towards a more frequent use of comprehensive molecular diagnostics in Swiss hospitals.

**Electronic supplementary material:**

The online version of this article (10.1186/s12911-018-0680-0) contains supplementary material, which is available to authorized users.

## Background

In recent years, molecular profiling based on high-throughput techniques has become an emerging practice in hospitals all over the world [1]. Decreased sequencing costs shifted the focus from traditional Sanger sequencing of a few specific genomic loci [2] to gene panels targeting a broader set of genes [3–5] and more comprehensive approaches, including whole-exome sequencing (WES) and whole-genome sequencing (WGS) [6, 7].

These emerging technologies hold great promise, in particular in regard to the identification of therapies for cancer patients [8]. Cancer is one of the leading causes of death in developed countries [9]. Despite decades of research the mechanisms underlying carcinogenesis are not fully understood and suitable treatment is not always available. Therapies are typically active only in a limited number of patients. Further, they are only successful for a short period of time before the tumor develops resistance mechanisms that lead to further disease progression [10–12]. Since tumors evolve via complex genetic changes [13], genomic profiling of a tumor allows the prediction of targeted therapies that are more likely to be active [6].

In recent years, an increasing number of molecular targets and corresponding drugs have been identified. Prominent examples are BRAF mutations in metastatic melanoma [14] and HER2 overexpression in breast cancer, which can be targeted by specific kinase inhibitors or monoclonal antibodies, e.g. vemurafenib and trastuzumab, respectively [15, 16]. Consequently, the first molecular diagnostics initiatives using comprehensive next-generation sequencing (NGS) were launched 3–4 years ago [17], initially mainly in the US (MD Anderson [18], Mayo Clinic [19], Weill Cornell [20]), but recently also in Europe (DKFZ/NCT [21]). In these initiatives, WES is used to comprehensively analyze the protein-coding genes of the tumor genome. This allows us to detect not only cancer type specific alterations, but also mutations common in other cancer types, or mutations with associated therapies that are currently in clinical development. These efforts personalize cancer treatment with a focus on suggesting therapy options for late-stage patients for whom standard treatment is no longer effective. In this setting, reported success rates of proposing therapies are at approximately 30% [19, 21]. When standard therapy options are also included (for instance when applied as part of routine diagnostics), therapy recommendations can be made for approximately 60% of the patients [22]. At first glance, these numbers appear low; however, there are multiple factors influencing the actual success rate when using personalized molecular diagnostics. First of all, it can be expected that with more known targets these numbers will increase. In addition, one major hurdle for precision medicine is drug access [19], as therapies beyond standard of care are often not available, i.e., not approved or not covered by health insurances, and access to clinical trials is limited. Furthermore, currently comprehensive molecular diagnostics is mainly applied to end-stage cancer patients progressive on standard therapies. This limits the number of therapies left for suggestion. Also, rapid health deterioration or death is the main reason for these patients not receiving molecular diagnostics-based therapy. Finally, patient drop-out as well as limited awareness of therapy options are a major issue impeding the measurable success of molecular diagnostics [23].

In Swiss hospitals, targeted deep amplicon sequencing mainly based on the Ion Torrent sequencing platform [24] is currently the established standard for molecular diagnostics. In targeted sequencing, not the complete exome but rather an a priori defined set of genes is analyzed in order to reduce sequencing costs and analytical complexity [3–5]. Gene panels need to be updated regularly to follow the discovery of new targeted therapies. In the last 2 years, 20 new oncological therapies have been approved by regulatory authorities in Switzerland [25]. However, by design gene panels used in the clinic (e.g. the different Ion Torrent Oncomine gene panels [26]) only include the most common targetable genes matching approved therapies. Thus, to identify therapy options beyond standard of care more comprehensive sequencing technologies such as WES or WGS may be beneficial.

Regardless of the sequencing technology, high-throughput sequencing-based molecular diagnostics need a reliable framework for annotation and clinical interpretation of genetic variants [27, 28]. Currently, clinical interpretation is mainly performed manually and thus very time-consuming. Also, it is prone to miss very recent or less well-known treatment options. In addition, available databases for drug-gene interactions are either lacking the level of manual curation necessary for reliable variant interpretation [29–31] or are manually curated but cover only a subset of available targets and therapies (e.g. the current build of mycancergenome.org contains information on 823 genes and 24 cancer types [32]). While the major focus of molecular diagnostics is to propose a suitable therapy, the amount of time used to reach such a proposition is of importance as well. For instance, patients that showed progressive disease on all standard therapy lines usually have only limited survival prospective. Currently, the turnaround time of comprehensive molecular diagnostics workflows is typically about 3–12 weeks [20, 33]. This needs to be reduced considerably in order to make routine clinical application feasible, particularly for end-of-treatment line patients.

Clinical interpretation has to be based on an accurate picture of the genetic landscape of the tumor and its origin. Thus, reliable variant calling is an important part of each molecular profiling workflow. Benchmarks on existing variant calling pipelines indicate that the performance of variant detection methods can vary significantly [34–36]. Each tumor presents a unique set of genetic alterations that were involved in carcinogenesis. Moreover, tumors do not evolve homogeneously, but as subclonal populations. Each subclone has certain variants not shared with other clones of the same tumor, which is challenging for the accurate variant identification. As a result, choosing the right set of tools and parameters for a particular analysis is difficult. These multifaceted challenges, which reach across different fields of expertise, require an interdisciplinary approach that combines different areas of knowledge and experience to advance the treatment of cancer patients [17, 28]. Clinical staff and bioinformaticians need to collaborate to identify common criteria and goals to enable a meaningful analysis and interpretation of genetic variants. In addition, a definition of general rules for data sharing between hospitals and computational groups is required to enable a smooth and secure data exchange.

With the aforementioned challenges in mind, over the last 2 years we combined the bioinformatics expertise of the ETH technology platform NEXUS Personalized Health Technologies (NEXUS) with the clinical expertise of the University Hospitals in Zurich and Basel to design and implement a workflow for molecular diagnostics of cancer patients. We aimed at supporting treatment decisions by comprehensively analyzing the genetic landscape of tumors to link actionable genetic variants with therapy recommendations. Tumor biopsies were collected at the hospitals and then underwent high-throughput sequencing by WES, WGS, RNA sequencing (RNA-seq), or gene panels. The tumor samples were analyzed to identify targetable genetic variants, which were further prioritized based on their clinical significance. All findings were summarized in a concise clinical report and discussed in a multidisciplinary molecular tumor board with the goal to recommend treatment options to the treating clinician.

## Methods

**Fig. 1:**
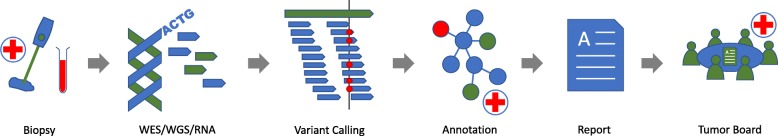
SwissMTB molecular diagnostics workflow. DNA (and RNA) is extracted from a tumor biopsy (and paired control tissue, e.g., blood) and sequenced. The resulting data is analyzed to detect genetic alterations (only in the tumor sample), which are associated with potential therapy options. Suitable therapies and clinical trial options are summarized in a clinical report, which is returned to the clinician and discussed in the molecular tumor board

As depicted in Fig. 1, SwissMTB is a collaborative effort of clinicians and bioinformaticians. A tumor biopsy and preferably a matched normal control sample (e.g., blood) undergoes DNA and possibly RNA extraction, followed by sequencing. Afterwards, the samples are analyzed with regard to somatic alterations, i.e., genetic changes that appear in the tumor, but not in the normal tissue. If tumor RNA-seq data is available, gene expression levels are determined and compared to a suitable reference cohort, e.g. TCGA Skin Cutaneous Melanoma [37], to identify over- and underexpressed genes. Both genetic and transcriptomic changes are then associated with possible therapy options. Relevant therapies are summarized in a clinical report that is discussed in an interdisciplinary tumor board.

In our pilot study, we included patients from University Hospital Zurich and University Hospital Basel. In Zurich, samples from stage IV melanoma patients progressive on standard therapy were analyzed using WES, WGS, and, when feasible, RNA-seq. In Basel, samples of various cancer types were analyzed using different Ion Torrent amplicon panels. Our main objective is to identify targetable aberrations and to recommend new therapy options for patients. Therefore, somatic variants such as single nucleotide variants (SNVs), small insertions and deletions (InDels), and copy number variants (CNVs) were of particular interest. In addition, the overall mutational burden as an indicator of response to immunotherapy in melanoma [38] is determined based on WES. Furthermore, WES enables inference of the patient’s HLA-I type as a treatment co-determining factor, which is required for certain vaccination trials [39, 40]. RNA-seq is used to determine differentially expressed genes as well as to validate targetable genomic variants, in particular copy number changes.

### A: Sample extraction and sequencing

Tumor tissue and, if possible, a normal control sample (blood) of the patient are collected at the hospital. For the SwissMTB pilot study fresh frozen samples (WES/WGS/RNA-seq) as well as formalin-fixed paraffin embedded (FFPE) samples (gene panels) were used. Integrated quality control steps ensure that genetic aberrations and potential therapies are only inferred for samples and regions passing previously defined quality thresholds.

DNA was extracted from the samples and prepared according to the respective sequencing protocols. Panel sequencing was performed directly at the pathology department of the University Hospital Basel. WES and WGS sequencing were performed in external sequencing facilities, either the Genomics Facility Basel or the Functional Genomics Center Zurich. To save costs, in our pilot study we performed a combined analysis of deep coverage WES (approx. 120x-230x tumor coverage) for somatic variant calling and low coverage (approx. 4x-13x tumor coverage) WGS for CNV calling. For technical details such as used panel type refer to Section “Results”.

### B: Bioinformatics analysis

**Fig. 2:**
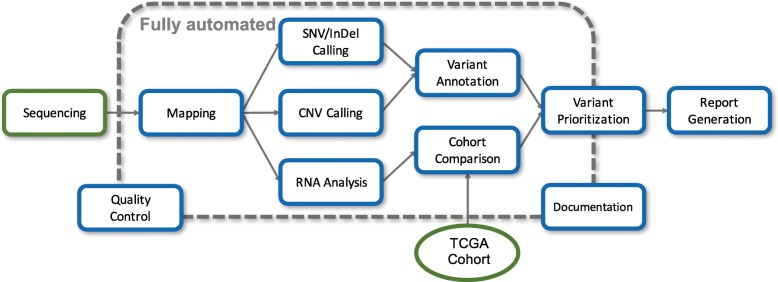
Overview of the SwissMTB bioinformatics analysis workflow. The reads generated by the sequencer are first mapped to the human reference genome. Afterwards, somatic variant and copy number variant calling is performed. Variants are annotated and then prioritized according to clinical relevance. RNA-seq based gene expression levels are compared to publicly available tumor sample cohorts. The findings are summarized in a clinical report. All steps from mapping to prioritization are fully automatized using a Snakemake-based pipeline. Selecting variants and therapies for the report is currently mainly manual work. All steps are documented and quality controlled, partly based on built-in routines in the analysis pipelines

After sequencing, the resulting short DNA and RNA fragments (called *reads*) were analyzed in order to identify genomic and transcriptomic aberrations. In the following, we briefly describe the bioinformatics analysis (Fig. 2).

#### DNA analysis

WES, WGS, and panel analysis mainly differ in their pre-processing steps. The annotation and clinical interpretation of variants is independent of the sequencing type. First, the short DNA reads generated by the sequencing machine are mapped to the human reference genome. The resulting mapping is post-processed and used as the basis for somatic variant (SNPs and InDels) and CNV calling (based on WGS). The pipeline for WES/WGS analysis from reads to unannotated variant calls is based on the framework described in [41], employing the Snakemake workflow environment [42]. Briefly, we follow the GATK best practices for variant calling [43]. Instead of using a single somatic variant caller we use a combination of three callers, namely MuTect [44], VarScan2 [45], and Strelka [46]. Only variants reported by at least two callers are considered, in order to identify variants with greater confidence and to reduce the number of false positive calls. For CNV calling on WGS data we use BIC-seq2 [47]. If WGS is not available, we use EXCAVATOR [48] on WES data. Variant calling on panel data depends on the sequencing platform. For Ion Torrent data we rely on variants called by Ion Reporter [26], the software suite accompanying Ion Torrent. For Illumina data, we use the variant callers mentioned above, i.e., MuTect, VarScan2, and Strelka.

#### RNA analysis

If available, RNA-seq data is processed based on the TCGA mRNA-seq Pipeline for UNC data [49], using STAR [50] to align RNA reads to the human reference genome. Typically, RNA-seq is only performed on tumor tissue, since it is often infeasible to obtain an RNA sample extracted from corresponding normal tissue. In order to determine if the expression of a gene is altered, we compare it to the gene’s expression in the most appropriate publicly available reference cohort. In our pilot study, we used either the TCGA Skin Cutaneous Melanoma [37] (SKCM) or the TCGA Uveal Melanoma [51] (UVM) cohort. Normalized gene expression values for those cohorts were downloaded from the Broad Institute TCGA GDAC Firehose website [52]. Targetable genes are considered for inclusion in the clinical report if they are either over- or underexpressed. Furthermore, gene expression is also used in the prioritization of genomic variants. For instance, a gene deletion detected by WGS or WES would be supported by RNA-seq if the observed gene expression is low or the gene is not expressed at all.

#### Annotation and prioritization

The number of observed somatic variants differs greatly depending on the tumor type [53]. However, the number of potentially interesting variants for a patient is usually too large to be processed manually, particularly within the desired short time-frame for generating a clinical report. Thus, the identified variants are annotated with information from databases such as COSMIC [54], dbSNP [55, 56], and ClinVar [57]. Additionally, we query cBioPortal [58, 59] to inform on the incidence of each variant across cancer types. Further, we annotate overall mutation type (e.g., missense or nonsense mutation, splice site mutation etc.), likely functional impact, and associated pathways. These types of information help to prioritize variants with respect to their significance for tumor development or treatment resistance. For instance, in colorectal cancer new mutations in the MAPK signaling pathway can confer resistance against combined RAF/MEK therapy by sustaining the activity of the pathway [60].

Furthermore, we query DGIdb [29, 61, 62] to get the drug-gene interactions reported in a collection of 30 databases, of which several are expert-curated (e.g. MyCancerGenome [63]). The more databases support an interaction, the higher the priority of the drug. In addition, we query all possible drugs at Swissmedic [25], the Swiss regulatory authority for drugs and medical products. Of highest priority are drugs that are available in Switzerland. Finally, based on drug-gene interactions identified in DGIdb we collect associated clinical trials at ClinicalTrials.gov. Clinical trials are important in two regards: First, completed trials inform on the suitability of a therapy and its confidence level in the report. Second, open clinical trials make therapies available to the patient, if the patient is eligible for the trial. Trials that explicitly require the observed variant for therapy eligibility are of particular interest.

The combination of the aforementioned information is essential for the generation of a clinical report reliably summarizing all relevant therapies for the treating clinician. Until this point, all steps are automated, and depending on sequencing depth the bioinformatics analyses takes approximately six hours. The main bottleneck regarding turnaround time is the manual generation of the clinical report, which is described in the following section.

### C: Clinical report

The clinical report is a concise summary of relevant therapies identified based on the genetic and transcriptomic landscape of the tumor. It is the basis for discussion in a molecular tumor board and informs on therapy options arising from the molecular alterations present in the tumor. Each recommended therapy is associated with an identified variant, and the relevance and reliability of each therapy is assessed based on evidence in the literature, such as previous preclinical studies and clinical trials. The clinical report is intended to only present the relevant subset of variants found in a tumor. This subset is derived based on the knowledge at the time of analysis and is thus likely to change over time, for instance if new targets are identified or therapies become Swissmedic approved. Therefore, each report is accompanied by an addendum listing all identified aberrations.

The clinical report is structured such that the results are communicated to the tumor board as purposeful as possible [64]. It has an overview page, presenting the most important information regarding therapy recommendations, accompanied by various sections with relevant details (Additional file 1).

**Fig. 3:**
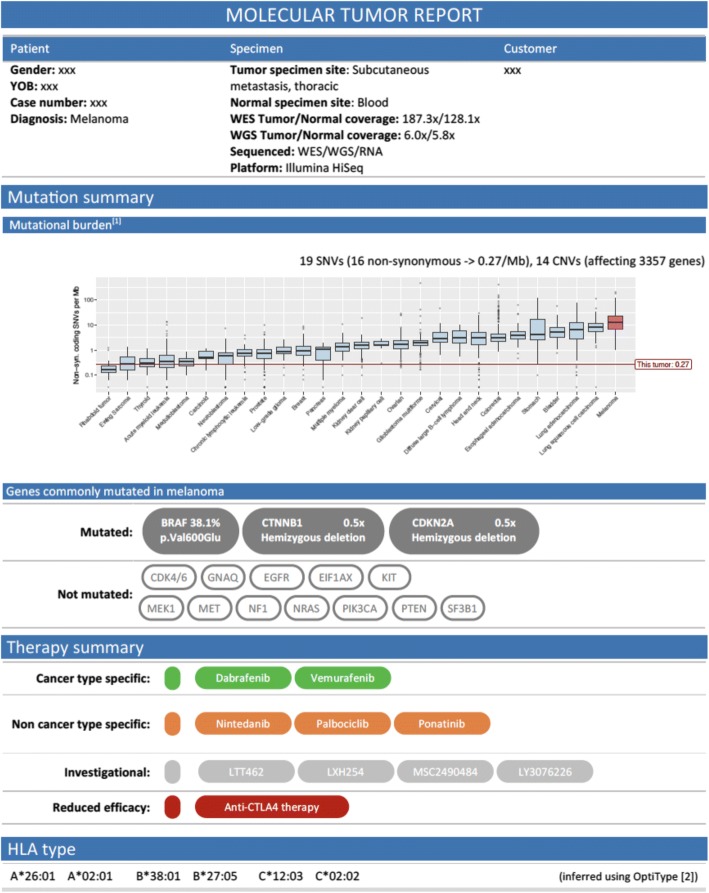
The overview page of an example clinical report including the categorization of therapies into cancer type specific, off-label (non cancer type specific), investigational, and possibly contraindicated therapies. We indicate the mutation status of commonly mutated genes, visualize the mutational burden of the patient, and inform on the patient’s HLA type

The overview page (Fig. 3) contains basic data on the patient, the tumor sample, and the treating clinician. It further summarizes the proposed therapy options, and presents results on mutational burden and HLA-I type. In addition, it gives an overview of the mutational status of genes frequently described as mutated in the given cancer, to allow the clinician to easily assess whether important genes are mutated or not. For instance, the status of BRAF in melanoma (mutations prevalent in 40–50% of all cases [14]) or ALK in lung cancer (rearrangements prevalent in 4–5% of all non-small cell lung cancers [65]) influences eligibility for clinical trials. The list of important genes is derived either by personal communication with the responsible clinician or based on MyCancerGenome [63] and relevant literature.

**Fig. 4:**
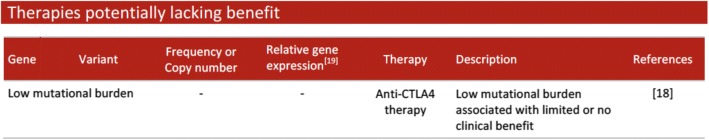
Report section for therapies potentially lacking benefit. Gene name and variant type, as well as observed frequency, copy number, or gene expression are presented to indicate resistance-causing events. Furthermore, details on the affected therapy, as well as a brief description of the finding and literature support are provided

In addition to therapy recommendations, also indicating possible drug resistances is of interest. For instance, various TP53 mutations have been reported to confer resistance to platinum-based chemotherapy in ovarian cancer [66]. Such findings are reported in a separate section (entitled “Therapies potentially lacking benefit”) and are also highlighted on the overview page. In our example, the low mutational burden indicates only limited benefit of Anti-CTLA4 therapy (Fig. 4) [38].

**Fig. 5:**
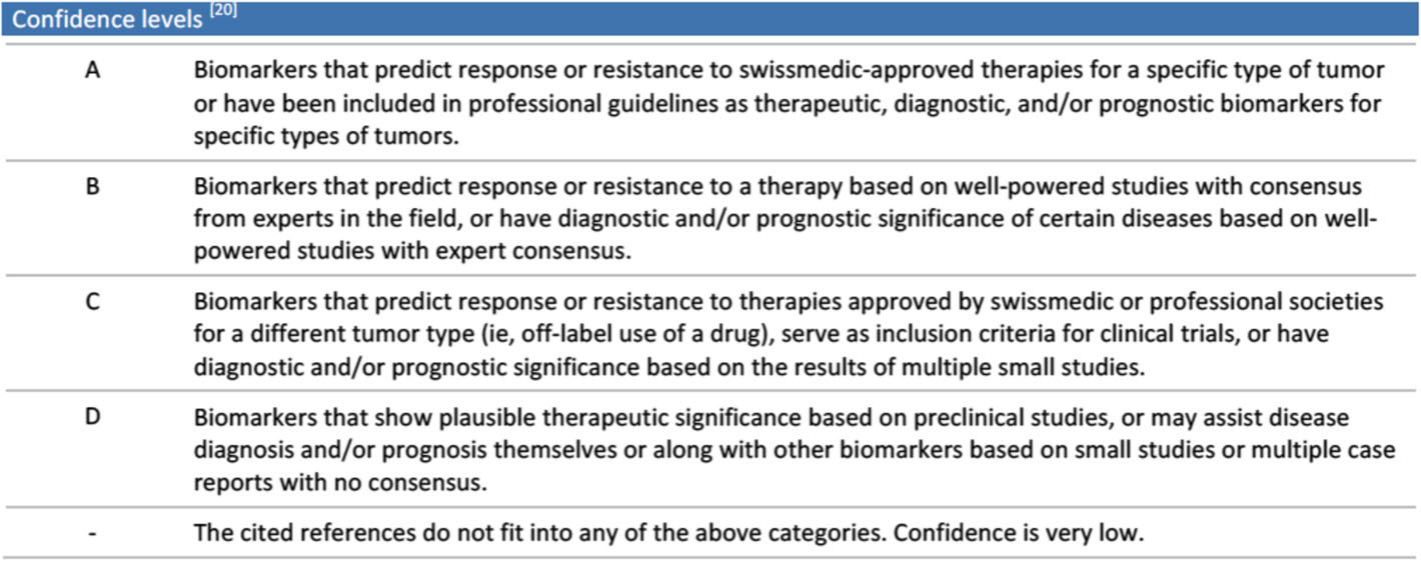
Therapy recommendation confidence levels, based on categorization by the Association for Molecular Pathology, American Society of Clinical Oncology, and College of American Pathologists [67]

We categorize findings into different levels of confidence that are used to assess the relevance of the potential therapy. For categorization, we follow the guidelines in [67] (refer to Fig. 5 for the confidence level overview).

Recommended drugs that are Swissmedic approved have the highest confidence level (level A), followed by those that have proven successful for treatment in several well-powered clinical studies (preferably phase 3). Drug targets that are Swissmedic approved for a different type of cancer or have been tested in smaller studies for the cancer type at hand have lesser confidence (level C). Finally, targets that have only been tested in preclinical studies are in the lowest confidence category (level D). This way the applicability of a recommended therapy can be assessed easily. In addition, we list the literature support for each included drug-gene interaction to allow a detailed assessment of the reasons why this particular therapy is recommended.

**Fig. 6:**
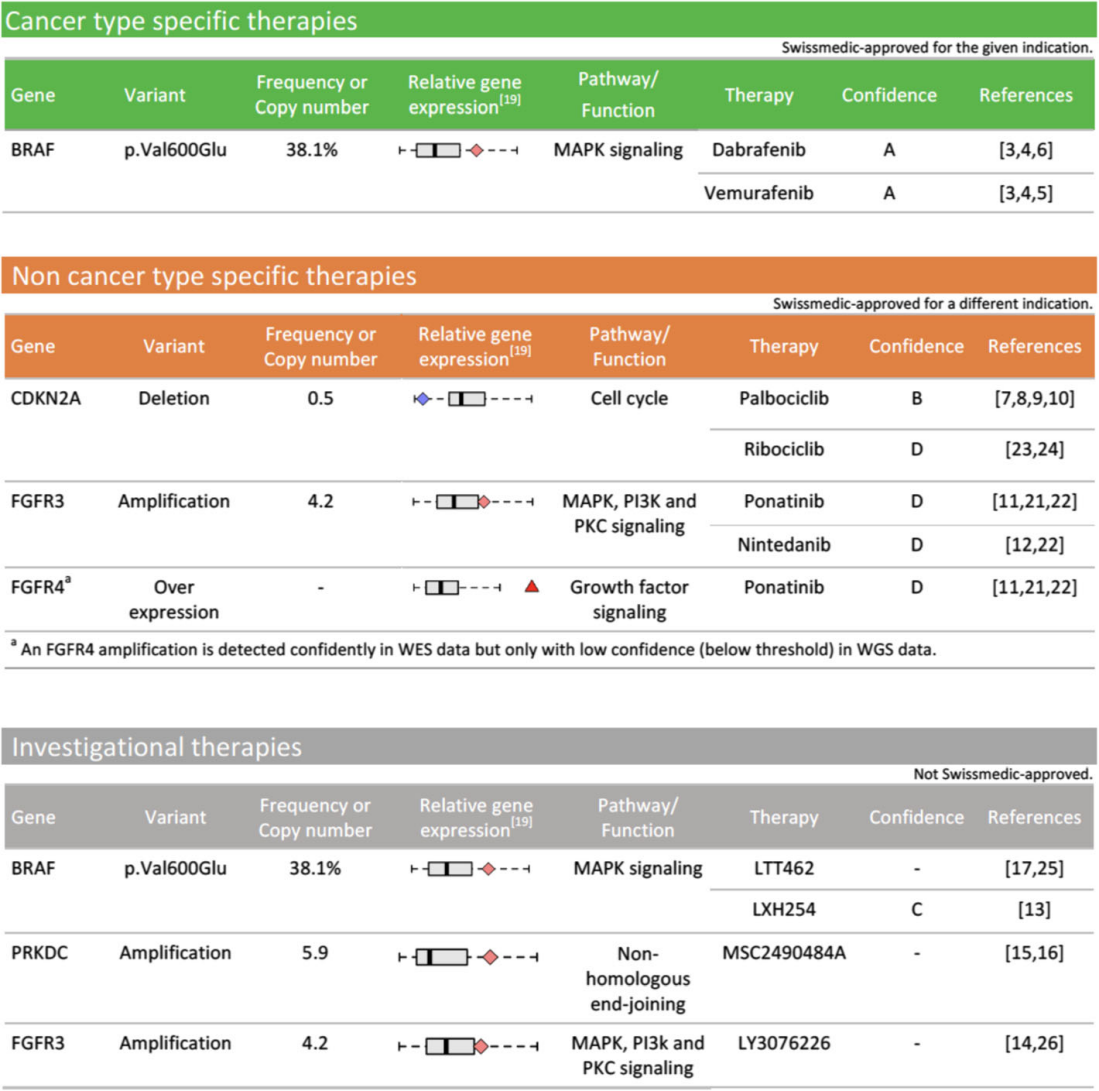
Example for therapy categories for a melanoma patient presenting (among other mutations) several copy number mutations, an FGFR4 overexpression, and a BRAF V600E point mutation, identified based on WES, WGS, and RNA-seq data. Each table is structured as follows: Column “Gene” shows the name of the affected gene. Column “Variant” either contains the exact amino acid change resulting from a point mutation, or states the copy number change (amplification or deletion), or presents the change observed by the RNA-seq analysis (e.g. overexpression). Column “Frequency or Copy number” presents the variant frequency of point mutations (in percent), or presents the copy number observed for the affected gene. Column “Relative gene expression” includes the boxplot that shows the expression of the particular gene in comparison to the TCGA cohort of the same cancer type. For ease of interpretation, the different types of boxplot are explained in the “Guide section” of the clinical report, Additional file 1. Column “Pathway/Function” gives details on the functions of a gene. Column “Therapy” shows the name of the drug with a potential drug-gene interaction, while columns “Confidence” and “References” present the confidence level and the literature support of the drug-gene interaction, respectively

In the report, therapies are grouped into different sections (Fig. 6), namely “cancer type specific therapies” (Swissmedic approved for the given indication), “non cancer type specific therapies” (Swissmedic approved for a different indication), and “investigational therapies” (not Swissmedic approved). The different sections contain the most relevant information for each recommendation: gene and drug name, observed variant, variant frequency or copy number, associated pathway, variant effect (if known, for instance gain-of-function or loss-of-function as indicated in OncoKB [68]), confidence level, and literature support. Further, as exemplified in Fig. 6, the relative gene expression of the mutated gene in comparison to the TCGA cohort of the same cancer type is shown in a separate column (the gene expression plots are explained in the report glossary, Additional file 1).

**Fig. 7:**
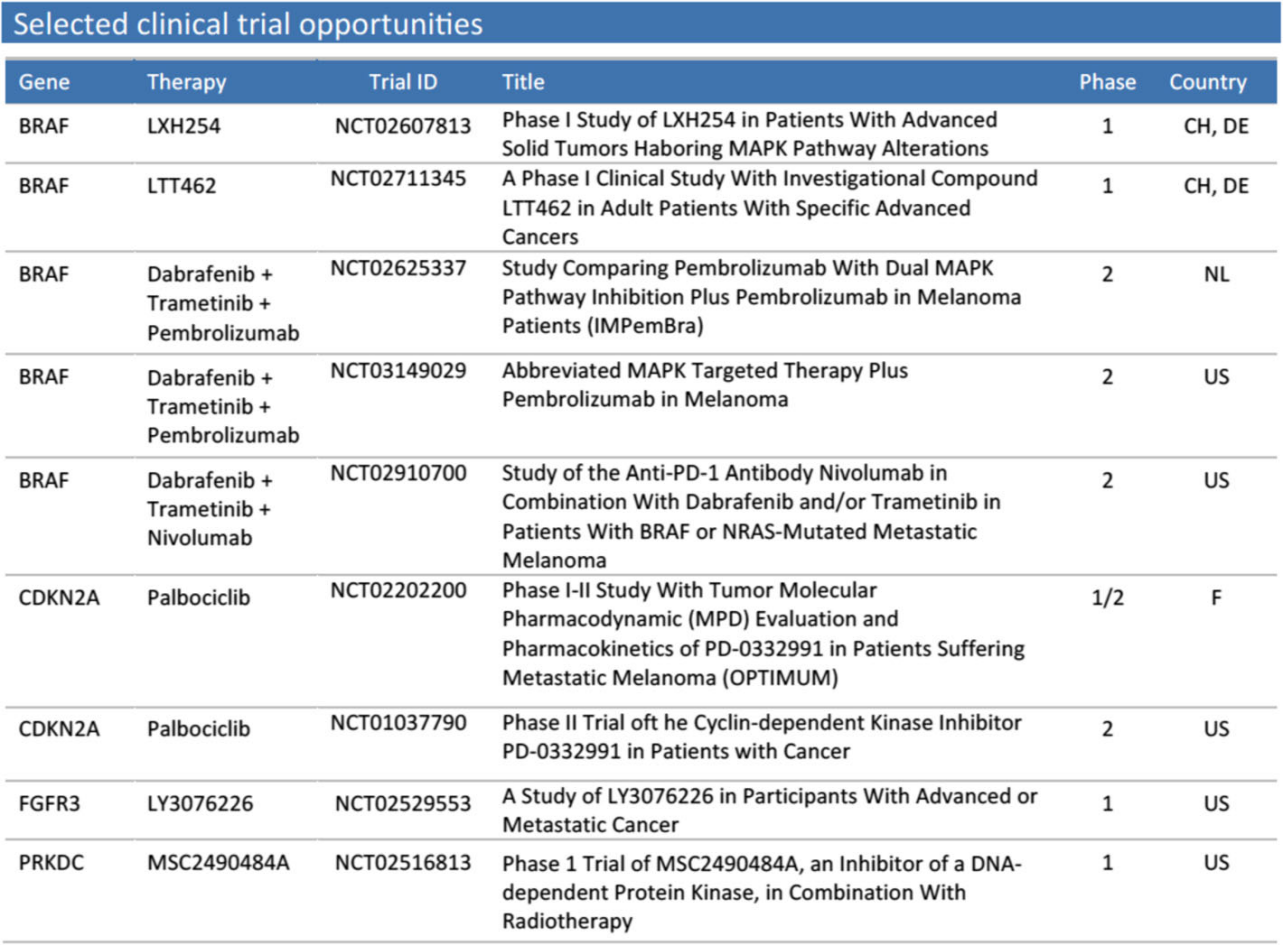
Example for clinical trial options presented in a clinical report

Finally, not only therapies are recommended to the treating clinician, but also potentially relevant open clinical trials (refer to Fig. 7 for an example table). We prefer trials of higher phase and located in or near Switzerland, and give a brief overview of each trial by including study title, trial phase, and location. Ultimately, suitability of a trial has to be decided by the clinician, because we do not have access to all information necessary to assess a patient’s eligibility.

The BRAF mutation observed in our example patient illustrates how the clinical report can be utilized to facilitate clinical decision making. BRAF V600E is a well-known therapy target in melanoma and thus assigned the highest confidence. As shown in Fig. 6 in the green table, Swissmedic approved therapies are available (dabrafenib and vemurafenib). However, also investigational therapies targeting BRAF are possible, namely LTT462 and LXH254. Both compounds are not yet Swissmedic approved and have only been tested in a few studies, but they might become relevant therapy options if the patient has already received (and progressed on) other therapies. As shown in Fig. 7, for both LTT462 and LXH452 clinical trials are recruiting in Switzerland, such that the compounds might indeed be accessible to the patient. This example shows the tight link between the different sections of the clinical report: at first glance investigational therapies might seem rather irrelevant and hardly accessible, but the clinical trials section directly shows whether the drug might nevertheless be available. It also highlights the advantages of comprehensive sequencing: only focusing on well-known targets cannot provide information on newer and less known therapy targets. Since tumors often become resistant to the applied therapies, pointing out alternatives that might work on these resistant tumors may be important for pre-treated patients.

## Results

Up to now, 22 patients progressive on standard treatments have been included in the SwissMTB study. From the dermatology department at the University Hospital Zurich we prospectively analyzed eleven patients with metastatic cutaneous, uveal, and mucosal melanoma (Table 1). These patients were sequenced based on WES accompanied by WGS (except for the first patient for whom only WES was performed). For five of the most recent patients we also performed RNA-seq to identify transcriptomic aberrations. In three of these patients, gene expression results affected therapy recommendations. From the University Hospital Basel, we analyzed eleven patients with various cancer types (Table 2). These samples were analyzed in retrospect, after the actual therapy had been applied.

**Table 1:** Overview of patients analyzed based on comprehensive sequencing

Patient	Cancer Type	Sequen-cing	No. of actionable variants	No. of therapies (cancer-type specific, off-label, investigational)	No. of therapies lacking benefit	Comments
1	Cutaneous Melanoma	WES	13	12 (2,4,6)	NA (not yet part of workflow)	Patient died before the report could be delivered. Based on an observed PTCH1 amplification reported in the SwissMTB analysis, vismodegib would have been considered as treatment.
2	Cutaneous Melanoma	WES	13	19 (2,12,5)	5	Based on the high mutational load of this tumor anti-CTLA4 treatment was recommended. The patient was treated accordingly and showed a complete response.
3	Cutaneous Melanoma	WES/WGS	6	12 (3,4,5)	5	NRAS Q61K resistance variant identified and trametinib treatment recommended.
4	Uveal Melanoma	WES/WGS	3	7 (0,4,3)	5	Based on a PXR loss Taxol treatment was recommended. The patient was treated accordingly, but progressed after 2 months of therapy.
5	Cutaneous Melanoma	WES/WGS	5	8 (2,5,1)	3	Based on the high mutational load anti-CTLA4 treatment was recommended. The patient was treated accordingly with a combination of anti-CTLA4 and anti-PD1 therapy and showed partial response.
6	Mucosal Melanoma	WES/WGS/RNA-seq	3	7 (0,4,3)	3	At the time of report delivery, the condition of the patient did not allow any treatment.
7	Mucosal Melanoma	WES/WGS/RNA-seq	6	12 (0,7,5)	1	Patient died before the report could be delivered.
8	Uveal Melanoma	WES/WGS	4	10 (0,4,6)	1	Based on GNA11 Q209L variant treatment with sorafenib was decided but insurance did not cover drug costs.
9	Uveal Melanoma	WES/WGS/RNA-seq	4	10 (0,4,6)	1	Patient died before the report could be delivered.
10	Uveal Melanoma	WES/WGS/RNA-seq	5	11 (0,5,6)	1	Based on observed FGFR4 overexpression, ponatinib was recommended as off-label treatment. However, the patient died before the report could be discussed in the molecular tumor board.
11	Cutaneous Melanoma	WES/WGS/RNA-Seq	4	6 (0,4,2)	0	Based on observed MET overexpression the patient received crizotinib as off-label treatment.
Summary: median (IQR)			5 (2)	10 (4.5)	2 (3.5)	

*Abbreviations*: *IQR* Interquartile range

**Table 2:** Overview of patients analyzed based on panel data, only tumor samples were sequenced

Patient	Cancer Type	Panel type	No. of actionable variants	No. of therapies (cancer-type specific, off-label, investigational)	No. of therapies lacking benefit	Comments
12	Fibroblastic osteosarcoma	Oncomine Comprehensive Panel	0	0	0	Only germline variants detected in sample.
13	Head and Neck Squamous cell carcinoma	Cancer HotSpot Panel 2.0	12	12 (6,6,0)	0	Various damaging variants in genes of MAPK signaling pathway detected. Tyrosine kinase inhibitor treatment recommended.
14	Lung neuroendocrine carcinoma	Cancer HotSpot Panel 2.0	1	1 (0,1,0)	0	Based on FBXW7 D440Y variant mTOR inhibitor treatment recommended.
15	Ovarian serous carcinoma	Cancer HotSpot Panel 2.0	1	1 (1,0,0)	1	Based on observed TP53 V173 L resistance variant doxorubicin treatment recommended and platinum-based treatment discouraged. As indicated in SwissMTB analysis, progressive disease under carboplatin treatment.
16	Cutaneous melanoma	Cancer HotSpot Panel 2.0	2	3 (2,1,0)	0	Based on CDKN2A R80* loss-of-function variant off-label treatment with palbociclib recommended.
17	Chondrosarcoma	Oncomine Solid Tumor DNA Panel	10	9 (0,9,0)	3	Tyrosine kinase inhibitor treatment recommended. Patient was treated with pazopanib, response status unknown. Four resistance-associated variants found: ALK G1202R, KRAS S65 N, TP53 C275Y, and TP53 G245D.
18	Lung adenocarcinoma	Liquid Biopsy, Oncomine Solid Tumor DNA Panel	0	0	0	No tumor DNA contained in sample.
19	Lung adenocarcinoma	Liquid Biopsy, Oncomine Solid Tumor DNA Panel	1	0	1	Use of ALK inhibitors discouraged because of ALK G1202R resistance variant. As indicated in SwissMTB analysis, progressive disease under crizotinib treatment.
20	Lung squamous cell carcinoma	Oncomine Solid Tumor DNA Panel	1	1 (1,0,0)	1	Paclitaxel recommended based on observed TP53 R342* variant.Contradictive case, as a second TP53 R248Q variant is associated with increased chemotherapy resistance.
21	Neuroectodermal sarcoma	Cancer HotSpot Panel 2.0	0	0	0	No variants identified.
22	Cutaneous melanoma	Cancer HotSpot Panel 2.0	5	11 (1,9,1)	1	Multi-kinase inhibitor treatment recommended, cisplatin treatment discouraged based on TP53 R273S resistance variant.
Summary: median (IQR)			1 (3)	1 (6)	0 (1)	

Note that the SwissMTB analysis of these samples was performed retrospectively. *Abbreviations*: *IQR* Interquartile range

The SwissMTB analyses for the University Hospital Zurich were performed before treatment. Thus, for some patients the molecular findings could influence treatment decisions. Unfortunately, similar as in other molecular tumor board approaches (e.g. [19, 69]), half of the patients experienced rapid health deterioration, such that for only six patients SwissMTB treatment recommendations could be discussed in the molecular tumor board. Namely, from the eleven patients presented in Table 1, patients 1, 6, 7, 9, and 10 died or were too far declined in health before the findings could be presented to the clinicians. Results from the six remaining patients have been discussed, and for five patients, namely patients 2, 4, 5, 8, and 11, the SwissMTB findings indeed influenced the treatment decision.

To be more specific, for patient 2 (refer to Table 1) the WES analysis showed that the sequenced tumor material had a very high mutational load with 826 non-synonymous protein-coding mutations. A high mutational load above 100 non-synonymous coding mutations has been shown to be predictive of positive response to ipilimumab therapy in melanoma [38]. Based on this finding and the recent approval of the combined checkpoint blockade of anti-CTLA4 and anti-PD1 therapy that results in higher response rates than single anti-CTLA4 treatment [70], combination treatment with ipilimumab and nivolumab was started. The patient experienced complete tumor regression after 2 months and continued on immunotherapy treatment, where he stayed tumor-free for 8 months. Furthermore, the patient’s tumor harbored amplifications of BRAF, EGFR, MET, and CDK6, which provides a rationale for this tumor’s acquired resistance to the triple BRAF/MEK/CDK4&6 inhibitor treatment applied before sequencing [71, 72].

For patient 3 (Table 1) the results showed an NRAS Q61K activation resistance variant, which leads to reactivation of the MAPK pathway in the presence of BRAF inhibitors. Trametinib, which inhibits the downstream MEK kinase, was therefore suggested as a matching drug on the report. However, it is known that the in vitro response of double NRAS and BRAF mutated cells to MEK inhibitors is heterogeneous [73]. The tumor board therefore decided on the newly approved combination therapy of the checkpoint inhibitors ipilimumab with nivolumab. The patient progressed on this therapy, which is in concordance with the low mutation rate reported by WES analysis and the associated contraindication of immunotherapy indicated in the report. At the time of manuscript submission, it was not yet decided whether the therapy will be switched to the alternatively proposed drug trametinib.

For patient 4 (refer to Table 1), who has a rare uveal melanoma, we reported the loss of the pregnane X receptor (PXR) as an actionable variant. This receptor binds chemotherapy agents such as taxanes and regulates drug metabolizing enzymes. PXR knockdown in cancer cells induces increased paclitaxel sensitivity and apoptotic cell death [74]. Also, in uveal melanoma paclitaxel treatment is known to induce stable disease in one third of patients [75]. The tumor board therefore decided to initiate treatment with paclitaxel. However, the patient progressed with new metastases after 2 months of treatment.

For patient 5 (Table 1) the report indicated a high mutational load. Therefore, the patient was treated with combined immune checkpoint blockade, which resulted in a partial response with most metastases regressing.

In patient 6 an amplification of the Src kinase LYN was detected in the WGS analysis. This indicates benefit of the LYN kinase inhibitors masitinib and bosutinib, which have been shown to be effective in cancer therapy [76, 77]. However, RNA-seq analysis showed that LYN expression is comparatively low with respect to the TCGA SKCM cohort [37]. Therefore, therapy with bosutinib and masitinib would likely have lacked benefit in this patient. This finding was included in the report. However, at the time of report delivery, the condition of the patient did not allow any treatment.

Based on the variant GNA11 Q209L identified in the uveal melanoma of patient 8 off-label treatment with sorafenib was decided by the tumor board. However, the insurance did not cover the therapy costs. Up to the time of manuscript submission no alternative treatment decision has been made.

For patient 10 FGFR4 overexpression was determined by comparing the patient’s gene expression to the TCGA UVM cohort [51]. Ponatinib and other pan-FGFR inhibitors are currently being investigated in clinical trials to determine their potential in treating FGFR aberrant cancers [78]. These treatments were included in the report. However, patient 10 died before report delivery, and thus these findings were not discussed in the tumor board. For patient 11 we reported a significant MET overexpression with respect to the TCGA SKCM [37] cohort. In a recent study, Yang et al. [79] reported tumor shrinkage and clinical benefit in gastric cancer patients with MET overexpression on crizotinib treatment. Based on these findings, off-label treatment with crizotinib was started. Unfortunately, the patient progressed on this treatment and died after a few weeks.

In Basel, the SwissMTB workflow was applied retrospectively to samples of patients who had already been treated. Thus, the SwissMTB findings did not influence treatment decision, but we can compare recommended and discouraged therapies with the actual therapy choice. Table 2 summarizes the results of the SwissMTB analysis for the panel-based analysis of patients 12 to 22. Since patients 12, 18, and 21 presented no actionable variants we did not recommend any therapies. For all of the remaining eight patients we found variants with possible treatment relevance. Only patient 20 presented inconclusive results that would need further investigation. In four of the patients, namely patients 15, 17, 19, and 22, resistance mutations were identified. Notably, in two of these cases (patients 15 and 19) the patients had received therapies for which we found resistance mutations, and indeed the clinical follow-up showed lack of response to these therapies.

For patient 13 several damaging variants were identified in genes associated with the MAPK signaling pathway. Thus, in the report use of tyrosine kinase inhibitors was recommended, e.g. pazopanib or lenvatinib. However, at the time of treatment decision information on these molecular targets was not available to the clinician, and thus a targeted therapy was not considered. The patient instead received and progressed on three lines of therapy including carboplatin+paclitaxel chemotherapy and immunotherapy with nivolumab.

For patient 14 the SwissMTB molecular diagnostic identified a variant in the WD40 domain of the tumor suppressor gene FBXW7. We recommended mTOR inhibitor treatment, for instance with everolimus [80]. Mixed responses to this treatment have been reported in the literature [81]. However, low response was shown in particular in the presence of other simultaneous variants, for instance in KRAS. Since in the panel-based analysis the observed FBXW7 variant appeared as the only mutation, a therapy with mTOR inhibitors such as everolimus might be justified. For this patient no clinical follow-up data was available.

Patient 15, who had ovarian cancer, showed a TP53 V173 L mutation that is associated with a higher likelihood of platinum treatment resistance [66, 82]. The patient received the platinum-based chemotherapy carboplatin, and indeed the therapy lacked efficacy. In the SwissMTB report we instead recommended doxorubicin treatment, which was reported to be effective in in the presence of platinum resistance [83, 84].

Patient 16 received anti-CTLA4 immunotherapy, which resulted in a stable disease. In case of disease progression the SwissMTB report recommends off-label treatment with palbociclib to target the observed loss-of-function variant R80* in CDKN2A [85, 86].

Patient 17 presented four different resistance mutations, namely ALK G1202R, KRAS S65 N, TP53 C275Y, and TP53 G245D. Thus, in the SwissMTB report we discouraged the use of ALK inhibitors, cetuximab, and cisplatin treatment. Instead, we recommended treatment with tyrosine kinase and mTOR inhibitors such as pazopanib and everolimus based on several variants in genes associated to the MAPK signaling pathway and mTOR signaling pathway. The patient was treated with pazopanib; however, at the time of writing no information on treatment response was available.

Patient 19 presented a lung adenocarcinoma with an ALK G1202R variant, a mutation associated with general resistance against ALK inhibition [87–89]. As predicted based on the observed variant, the patient showed a progressive disease when treated with the ALK kinase inhibitor crizotinib.

The SwissMTB analysis of the lung squamous cell carcinoma of patient 20 presents an example of inconclusive results. Here, the only two identified variants were TP53 R342* and TP53 R248Q. Contradicting results are reported regarding the sensitivity or resistance to paclitaxel treatment in the presence of these variants [90, 91]. Thus, paclitaxel is indicated in the clinical report as possible therapy option that needs further investigation.

For patient 22 we identified TP53 R273S, a variant associated to cisplatin therapy resistance in a variety of cancer types [82, 92]. The patient showed a progressive disease under anti-CTLA4 and anti-PD1 immunotherapy. According to the SwissMTB molecular diagnostic other therapy options would have been off-label tyrosine kinase inhibition, e.g. with sunitinib or regorafenib, based on multiple variants in the MAPK signaling pathway and a KIT exon11 variant [93, 94].

## Discussion

The conducted pilot project only comprises a small number of patients and can thus only serve as a proof-of-concept to show the feasibility of the SwissMTB workflow in a clinical setting. Larger clinical trials including a control group are required to determine whether approaches based on comprehensive molecular tumor analysis offer significant clinical benefit to late stage cancer patients. Nonetheless, single patients showed clinical benefit from our SwissMTB approach.

In summary, we were able to recommend therapies for 19 out of 22 patients (86%), which exceeds the recommendation success rate of 60% generally reported in the literature [22]. As clinicians were directly involved in the SwissMTB workflow, our study did not suffer from limitations such as lack of awareness of available therapies. Thus, when the influence of factors like limited awareness of therapeutic options and patient drop-out is reduced, the actual benefit of molecular diagnostics might be higher than visible from numbers reported in the literature. For five out of eleven patients analyzed before treatment, the SwissMTB diagnostics influenced the treatment decision. Four of them received the recommended therapy (in one case the insurance did not cover the treatment costs), and two of these patients responded well. In addition, we identified clinically relevant variants in eight of eleven cases in the retrospective analysis based on panels. For six of these patients, SwissMTB proposed promising actionable targets and possible targeted treatments. Since most patients included in the pilot cohort progressed on their received standard therapy, these findings would have been relevant treatment alternatives or follow-up treatment options. Importantly, we also identified resistance variants for treatments that indeed proved ineffective in the clinical follow-up. Thus, for two patients analysed retrospectively, our analysis likely would have prevented a non-beneficial therapy. Overall, the results of this pilot cohort, despite being small and preliminary, indicate the benefit of the SwissMTB workflow. Further, the pilot study suggests an added value of integrating RNA-seq information, as gene expression results affected therapy recommendations in three out of the five cases with available RNA.

In Switzerland, SwissMTB is the first workflow for a molecular tumor board based on comprehensive sequencing techniques like WES, WGS, and RNA-seq. Other initiatives, e.g. at the Weill Cornell Cancer Center in New York [20], or at the DKFZ in Heidelberg [21], also implement comprehensive molecular diagnostics, although typically they are based on WES only [19, 20]. Only few cohort studies published so far include RNAseq-based analysis for direct treatment prediction [95, 96]. Importantly, since Swiss regulations differ from regulations in other countries, e.g. the US or Germany, molecular tumor board workflows from other countries cannot be applied straightforwardly in Switzerland. In contrast, SwissMTB was specifically designed to be used in Swiss clinical practice, and thus it adheres to the guidelines of the Swiss authorities.

Already today, many hospitals have implemented molecular tumor boards to discuss treatment decisions. However, these tumor boards almost exclusively look at NGS results from gene panel sequencing. In contrast, SwissMTB targets not only a limited set of genes, but offers a comprehensive view on the genomic and, additionally, transcriptional landscape of the tumor. Thus, it goes beyond the workflows currently implemented in the hospitals. Reasons why in Swiss hospitals gene panels are the de facto standard for NGS-based analyses are mostly the lower sequencing costs and straightforward variant interpretation. However, WES and WGS are more comprehensive and can provide a clearer picture of the mutational landscape of the tumor. For instance, important features such as the HLA type cannot be assessed with commonly used panels, but only with more comprehensive approaches. Similarly, tumor mutational burden, an important predictor of immune checkpoint therapy response, cannot be analyzed by most smaller routine panels. Routine diagnostics often lack the computational methods to filter and prioritize the often large number of variants identified in WES/WGS. Since SwissMTB provides the means to analyze and perform drug-matching of a large numbers of variants, our workflow paves the way towards a more frequent use of comprehensive molecular diagnostics in Swiss hospitals.

Another advantage of SwissMTB is its support of the simultaneous analysis of tumor and matched control samples. In Swiss hospitals NGS-based diagnostics is almost exclusively performed on tumor-only samples. However, we strongly encourage the use of a matched normal sample in order to directly call somatic aberrations and to decrease the risk of identifying germline variants. Recently, a study by Sun et al. [97] showed that clinically reliably somatic variant calling is also possible without a matched normal. However, the approach has various constraints such as requiring high sequencing depths of more than 500x, and thus is currently infeasible for WES and WGS in clinical practice. However, particularly for WES and WGS sequencing the number of germline variants would impede the search for tumor-relevant variants. Furthermore, investigating germline variants of a patient requires special ethical consideration. For panel sequencing the use of a matched normal control is advisable for the same reason. However, it is not yet common practice – and sometimes not even possible – to obtain such a sample. In our study, a matched normal sample was used for WES and WGS. In contrast, both panel-based analysis and RNA-seq were exclusively performed on tumor samples. As discussed below, this led to germline variant identification in one of the patients.

When we compare the overall results of the analysis based on comprehensive sequencing versus panel sequencing, we see that with panels generally less actionable variants are found, resulting in less therapy options and less insight into drug resistance and clinical trials. This effect can be observed independently of the panel type used, and further underlines the benefit of using comprehensive sequencing instead of targeted panels. Notably, the patients from University Hospital Basel presented various cancer types. Thus, compared to melanoma there might be less actionable variants and therapies available. For better comparability we only considered the patients with melanoma (two patients) or lung cancer (four patients) for a less biased second comparison. However, even on this limited number of patients overall less actionable variants and corresponding treatments were identified, namely a median number of one variant and one therapy, including standard therapies.

Notably, the patients from our cohort had already received a number of therapies before they entered our study. Even when excluding these therapies, still off-label and investigational therapies were recommended for all eleven patients analyzed with WES/WGS, and only for six patients analyzed based on panels. Each report based on comprehensive sequencing proposes a median number of ten such therapies linked to a median number of six trials per patient. Note that this number already represents a selected set of preferable trials (e.g. trials conducted in or near Switzerland, if those were available).

In the panel-based analysis, we identified actionable variants in eight of the eleven patients. For these eight patients, a median of two therapies was recommended per patient with a median of one off-label or investigational therapy linked to a median number of 2.5 clinical trials. There are several possible reasons for the lack of actionable variants in three of the eleven patients. In case of patient 18 (refer to Table 2) the amount of tumor DNA in the sample was not sufficient. This sample was taken by liquid biopsy, a technique that extracts cell-free circulating tumor DNA from the blood. Here, wild-type DNA is much more abundant than tumor DNA. Thus, the sequencing and bioinformatics analysis of liquid biopsy samples is even more challenging compared to tumor biopsy samples [98]. For patients 12 and 21 (Table 2) no variants could be identified, except for germline variants in patient 12. On the one hand, this shows the importance of a normal control sample to decrease the risk of identifying germline variants. On the other hand, both patient 12 and 21 presented rare tumor types (fibroblastic osteosarcoma and neuroectodermal sarcoma, respectively) for which few therapy targets are known. Patients with such rare tumor types might benefit from an analysis based on comprehensive WES/WGS, since panels might not contain the required targets.

In general, it would be advantageous to perform molecular diagnostics directly at the entry point in the clinic. This is particularly true for panel-based analyses. Patients at later stages that already received a number of standard therapies might need off-label and investigational therapy recommendations. The gene panel might not cover the relevant therapy targets and thus could fail to inform on additional therapy options, as can be observed in our study where for only six of the patients analyzed based on panels therapies beyond standard of care could be identified. However, Table 1 shows that also molecular diagnostics based on comprehensive sequencing would benefit from an earlier time of sequencing. To speed up the report delivery, we implemented a two-step procedure. A so-called level-1 report that includes unfiltered actionable variants and corresponding treatments is generated automatically without manual inspection. Thus, it is delivered to the clinicians and oncologists within one day after the sequencing has finished. The level-2 report, namely the clinical report including the filtered and manually inspected variants and treatments, is reported after approximately 4 weeks. However, even with this two-step procedure five of the ten dermatology patients died or presented a too severe health condition to allow starting new therapies before the report could be delivered. This stresses the importance of decreasing the analysis turnaround time. It also shows that patients can benefit most from molecular diagnostics if tumors are sequenced at diagnosis or before entering the last standard treatment line.

## Conclusions

In conclusion, molecular profiling based on high-throughput technologies is an emerging practice in hospitals all over the world that allows for the detection of genomic aberrations involved in carcinogenesis and therapy resistance. To the best of our knowledge, we introduced the first comprehensive high-throughput sequencing based molecular profiling workflow in Swiss clinics. In a collaboration of the NEXUS Clinical Bioinformatics Unit of ETH Zurich and the University Hospitals Zurich and Basel a workflow for tumor sample extraction, bioinformatics analysis, and clinical reporting has been implemented and tested in a pilot project involving 22 patients. We identified actionable targets in 86% of the patients, and in addition identified several resistance-causing variants. In five of eleven patients sequenced before treatment, SwissMTB findings influenced treatment decisions, which demonstrates the benefit of comprehensive sequencing based molecular diagnostics. We have shown that the close collaboration of clinicians and bioinformaticians enables the identification of new therapy options for end-of-treatment line patients. The wealth of information accessible with comprehensive high-throughput sequencing requires sophisticated analysis workflows, but allows for generating novel treatment suggestions. Thus, already today traditional Sanger sequencing-based diagnostic methods are being replaced by high-throughput NGS-based techniques.

Once decreasing sequencing costs will make reimbursement from Swiss health insurances for WES and WGS feasible, these methods likely will become standard diagnostic tools, not only for patients that have progressed on standard therapies but for every cancer patient entering the clinic. Our molecular diagnostics workflow provides a prototype that can form the basis for streamlined profiling and reporting required to enable such routine clinical use in Switzerland.

Possible extensions of the SwissMTB workflow include the integration of other molecular profiling technologies, such as proteomics and single cell sequencing [99, 100]. Proteomic analysis would provide information on the translated proteins in the tumor, thereby verifying variants identified on the genomic and transcriptomic level and additionally detecting post-translational modifications. Post-translational modifications, such as phosphorylation or histone modifications, have been shown to play a critical role in the development of a variety of cancer types [101, 102] and in drug resistance development. Their analysis would thus open a new window for therapy possibilities [103]. Single-cell sequencing provides more detailed information on the genetic makeup of the whole tumor tissue and its heterogeneous subclones [100, 104]. Currently, treatments target actionable variants that might be present in the majority, but not all tumor cells. Cells without the variant can survive and over time the tumor regrows. Single-cell sequencing could inform on treatments necessary to target all subclones of the tumor.

With declining sequencing costs, high coverage WGS will become feasible not only for research, but also in the clinics. This will enable the analysis of larger structural variants and eventually eliminate the need for WES or panel sequencing. Already today, for instance the 100,000 genomes project in the UK performs WGS of each sample at depths sufficient for variant calling [105]. In Swiss clinics currently panel sequencing is the standard diagnostic high-throughput technique as it is the least cost- and analysis-intensive, and covered by health insurances. However, decreasing costs for WES and WGS will enable reimbursement also for these techniques and shift the focus in routine diagnostics from panel sequencing to whole exome and ultimately whole genome analysis. Simultaneously, molecular diagnostics based on high-throughput techniques will become the tool of choice also for early diagnostics, not only to find last therapy options for patients that have progressed on all standard treatments. Applying comprehensive molecular cancer diagnostics already at the clinical entry point also has benefits for patients with unknown primary tumor. The genetic makeup of the the tumor can predict the tumor type, which improves the choice of suitable therapies [106].

One of the greatest challenges in the SwissMTB is the prioritization and clinical interpretation of genomic aberrations. This task is not yet fully automated and requires manual inspection of sometimes large numbers of drug-target combinations. Given the aspired short turnaround time, further work is needed to automate the literature search for prioritization of targetable variants. Here, one possible approach is the integration of text mining methods to pre-filter variants with respect to their importance and occurrence in the literature. Reducing the list of possible targets to only promising ones will greatly reduce the time and effort necessary to formulate a concise clinical report.

In addition to these technical developments, also the management and communication within the SwissMTB is a constant learning process. Bridging the gap between clinic and research and making the application of cutting edge technologies feasible for clinical use requires that experts from both clinical and bioinformatics world come together and discuss their needs for sufficient sample analysis. This involves the required analysis types and results, as well as the way findings are reported. Consequently, our clinical reports have evolved over time and will probably continue to evolve, based both on new technologies and on feedback from the treating clinician regarding the interpretability of analysis results. This iterative integration of new technologies and feedback into SwissMTB will also introduce new challenges. Various sources of information need to be summarized and presented in a concise and easily interpretable report to be useful for discussion in a tumor board. In the near future, the increasing number of profiling sources will require an interactive report rather than the traditional pdf document to prioritize information, to allow focussing on the most promising, and to access details on demand [64].

Because our workflow exclusively proposes therapies by summarizing the evidence seen in the genomic and transcriptomic landscape of the tumor, it is a new diagnostic tool for the clinician. Therefore, there is no need for a research-related patient consent. However, using such a consent form (as is common practice in Swiss hospitals) has the benefit to ensure that potential future research projects can access these data. For instance, with a growing number of sequenced patients a new valuable resource to interpret results develops. Given the appropriate consent, variants found in a certain tumor type, as well as chosen therapies and their outcomes, can form a resource which facilitates and improves therapy recommendations for new patients. This was for instance shown for acute myeloid leukemia in [107]. Naturally, also here the feedback from clinicians is essential, and data has to be handled carefully in order to ensure patient privacy.

Finally, approaching cancer therapy from a molecular perspective demands a new design for clinical trials. In recent years, so called basket trials emerged that stratify patients not by their disease but rather based on their genetic variants [69, 108]. These trials will be invaluable to assess the actual therapy potential of specific actionable variants across different cancer types. In addition, as was for instance shown in [109], they can serve to identify new molecular targets and thus broaden our understanding of tumor development and therapy.

## Additional file

Additional file 1: Example SwissMTB clinical report. (PDF 212 kb)

## Abbreviations

CNV: Copy number variant
DKFZ: Deutsches Krebsforschungszentrum
DNA: Deoxyribonucleic acid
FFPE: Formalin-fixed paraffin embedded
HLA: Human leukocyte antigen
InDel: Insertion and deletion
IRB: Institutional review board
NCT: Nationales Centrum für Tumorerkrankungen
NGS: Next-generation sequencing
RNA: Ribonucleic acid
RNA-seq: RNA sequencing
SKCM: Skin cutaneous melanoma
SNV: Single nucleotide variant
SwissMTB: Swiss Molecular Tumor Board
TCGA: The Cancer Genome Atlas
UNC: Uniform naming convention
UVM: Uveal melanoma
WES: Whole exome sequencing
WGS: Whole genome sequening
