# Supplementary material for: SwissMTB: establishing comprehensive molecular cancer diagnostics in Swiss clinics
Source: BMC Med Inform Decis Mak. 2018 Oct 29;18:89. doi: 10.1186/s12911-018-0680-0 (PMC6206832; doi:10.1186/s12911-018-0680-0)
Supplement: Supplementary file 1 — Example SwissMTB clinical report. (PDF 212 kb) [file 12911_2018_680_MOESM1_ESM.pdf]

MOLECULAR TUMOR REPORT

Patient	Specimen	Customer
**Gender:** xxx<br>**YOB:** xxx<br>**Case number:** xxx<br>**Diagnosis:** Melanoma	**Tumor specimen site**: Subcutaneous metastasis, thoracic<br>**Normal specimen site**: Blood<br>**WES Tumor/Normal coverage:** 187.3x/128.1x<br>**WGS Tumor/Normal coverage:** 6.0x/5.8x<br>**Sequenced:** WES/WGS/RNA<br>**Platform:** Illumina HiSeq	xxx

Mutation summary

Mutational burden[1]

19 SNVs (16 non-synonymous -> 0.27/Mb), 14 CNVs (affecting 3357 genes)

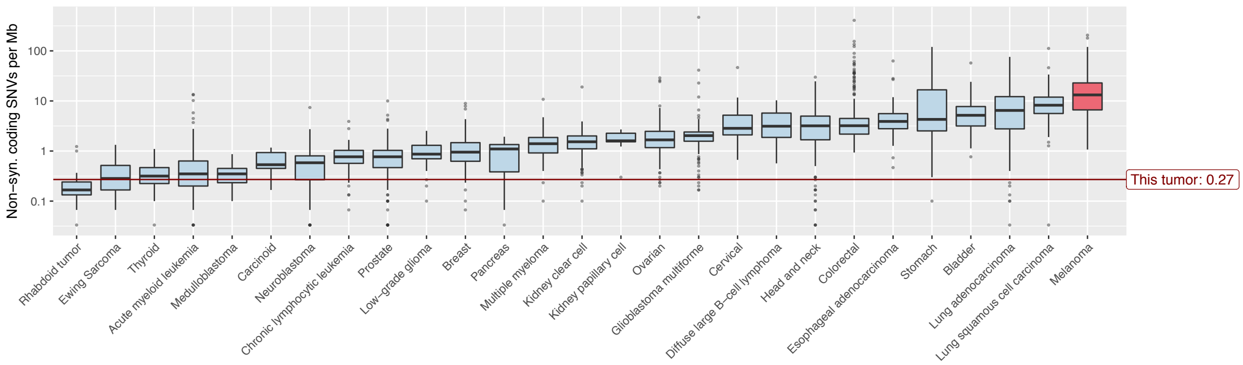

Genes commonly mutated in melanoma

Mutated: BRAF 38.1% p.Val600Glu | CTNNB1 0.5x Hemizygous deletion | CDKN2A 0.5x Hemizygous deletion

Not mutated: CDK4/6, GNAQ, EGFR, EIF1AX, KIT, MEK1, MET, NF1, NRAS, PIK3CA, PTEN, SF3B1

Therapy summary

Cancer type specific: Dabrafenib, Vemurafenib

Non cancer type specific: Nintedanib, Palbociclib, Ponatinib

Investigational: LTT462, LXH254, MSC2490484, LY3076226

Reduced efficacy: Anti-CTLA4 therapy

HLA type

A*26:01 A*02:01 B*38:01 B*27:05 C*12:03 C*02:02 (inferred using OptiType [2])

Cancer type specific therapies

Swissmedic-approved for the given indication.

Gene	Variant	Frequency or Copy number	Relative gene expression[19]	Pathway/ Function	Therapy	Confidence	References
BRAF	p.Val600Glu	38.1%	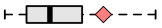	MAPK signaling	Dabrafenib	A	[3,4,6]
					Vemurafenib	A	[3,4,5]

Non cancer type specific therapies

Swissmedic-approved for a different indication.

Gene	Variant	Frequency or Copy number	Relative gene expression[19]	Pathway/ Function	Therapy	Confidence	References
CDKN2A	Deletion	0.5	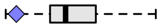	Cell cycle	Palbociclib	B	[7,8,9,10]
					Ribociclib	D	[23,24]
FGFR3	Amplification	4.2	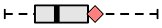	MAPK, PI3K and PKC signaling	Ponatinib	D	[11,21,22]
					Nintedanib	D	[12,22]
FGFR4[a]	Over expression	-	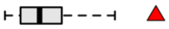	Growth factor signaling	Ponatinib	D	[11,21,22]

[a] An FGFR4 amplification is detected confidently in WES data but only with low confidence (below threshold) in WGS data.

Investigational therapies

Not Swissmedic-approved.

Gene	Variant	Frequency or Copy number	Relative gene expression[19]	Pathway/ Function	Therapy	Confidence	References
BRAF	p.Val600Glu	38.1%	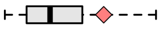	MAPK signaling	LTT462	-	[17,25]
					LXH254	C	[13]
PRKDC	Amplification	5.9	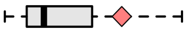	Non-homologous end-joining	MSC2490484A	-	[15,16]
FGFR3	Amplification	4.2	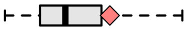	MAPK, PI3k and PKC signaling	LY3076226	-	[14,26]

Therapies potentially lacking benefit

Gene	Variant	Frequency or Copy number	Relative gene expression[19]	Therapy	Description	References
Low mutational burden		-	-	Anti-CTLA4 therapy	Low mutational burden associated with limited or no clinical benefit	[18]

Selected clinical trial opportunities

Gene	Therapy	Trial ID	Title	Phase	Country
BRAF	LXH254	NCT02607813	Phase I Study of LXH254 in Patients With Advanced Solid Tumors Haboring MAPK Pathway Alterations	1	CH, DE
BRAF	LTT462	NCT02711345	A Phase I Clinical Study With Investigational Compound LTT462 in Adult Patients With Specific Advanced Cancers	1	CH, DE
BRAF	Dabrafenib + Trametinib + Pembrolizumab	NCT02625337	Study Comparing Pembrolizumab With Dual MAPK Pathway Inhibition Plus Pembrolizumab in Melanoma Patients (IMPemBra)	2	NL
BRAF	Dabrafenib + Trametinib + Pembrolizumab	NCT03149029	Abbreviated MAPK Targeted Therapy Plus Pembrolizumab in Melanoma	2	US
BRAF	Dabrafenib + Trametinib + Nivolumab	NCT02910700	Study of the Anti-PD-1 Antibody Nivolumab in Combination With Dabrafenib and/or Trametinib in Patients With BRAF or NRAS-Mutated Metastatic Melanoma	2	US
CDKN2A	Palbociclib	NCT02202200	Phase I-II Study With Tumor Molecular Pharmacodynamic (MPD) Evaluation and Pharmacokinetics of PD-0332991 in Patients Suffering Metastatic Melanoma (OPTIMUM)	1/2	F
CDKN2A	Palbociclib	NCT01037790	Phase II Trial oft he Cyclin-dependent Kinase Inhibitor PD-0332991 in Patients with Cancer	2	US
FGFR3	LY3076226	NCT02529553	A Study of LY3076226 in Participants With Advanced or Metastatic Cancer	1	US
PRKDC	MSC2490484A	NCT02516813	Phase 1 Trial of MSC2490484A, an Inhibitor of a DNA-dependent Protein Kinase, in Combination With Radiotherapy	1	US

General Disclaimer

This report (or appendix) is based on an ongoing development project of NEXUS Personalized Health Technologies, ETH Zurich, and makes no promises or guarantees that a particular drug will be effective in the treatment of a disease in any patient. Furthermore, it makes no promises or guarantees that a drug listed under *Therapies potentially lacking benefit* will in fact provide no clinical benefit. Decisions on patient care and treatment must be based on the independent medical judgment of the treating physician. In accordance with the standard of care, all applicable information concerning a patient's condition has to be taken into consideration. A treating physician's decisions should not be based on a single test or solely on the information contained in this report.

Guide

Relative gene expression

Gene expression levels in the patient's tumor tissue are compared to gene expression level in a selected TCGA cohort of related tumor tissues. For a given gene, the box plot displays the distribution of the respective gene's expression within the cohort and the colored marker represents the expression in the patient's tumor sample. The markers are to be interpreted as follows:

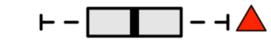	Over-expression	Expression in the patient's tumor is significantly higher than in the reference cohort.
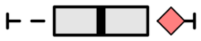	High expression	Expression in the patient's tumor is higher than in 75% of the samples in the reference cohort, but not significantly higher.
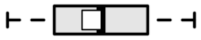	Normal expression	Expression in the patient's tumor is similar to the reference cohort.
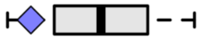	Low expression	Expression in the patient's tumor is lower than in 75% of the samples in the reference cohort, but not significantly lower.
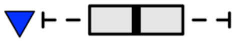	Under-expression	Expression in the patient's tumor is significantly lower than in the reference cohort.

For this report, the cohort comparison was performed using the TCGA melanoma cohort [19].

Confidence levels [20]

A	Biomarkers that predict response or resistance to swissmedic-approved therapies for a specific type of tumor or have been included in professional guidelines as therapeutic, diagnostic, and/or prognostic biomarkers for specific types of tumors.
B	Biomarkers that predict response or resistance to a therapy based on well-powered studies with consensus from experts in the field, or have diagnostic and/or prognostic significance of certain diseases based on well-powered studies with expert consensus.
C	Biomarkers that predict response or resistance to therapies approved by swissmedic or professional societies for a different tumor type (ie, off-label use of a drug), serve as inclusion criteria for clinical trials, or have diagnostic and/or prognostic significance based on the results of multiple small studies.
D	Biomarkers that show plausible therapeutic significance based on preclinical studies, or may assist disease diagnosis and/or prognosis themselves or along with other biomarkers based on small studies or multiple case reports with no consensus.
-	The cited references do not fit into any of the above categories. Confidence is very low.

Appendix

For an overview of all identified mutations please refer to appendix xxx.

References

[1] Alexandrov et al., "Signatures of mutational processes in human cancer", Nature, 500:415-421 (2013).

[2] Szolek et al., "OptiType: precision HLA typing from next-generation sequencing data.", Bioinformatics, 30(23):3310-6 (2014).

[3] Ascierto, Paolo A., et al. "The role of BRAF V600 mutation in melanoma." J Transl Med 10.85 (2012): 10-1186.

[4] Tsao, Hensin, et al. "Melanoma: from mutations to medicine." Genes & development 26.11 (2012): 1131-1155.

[5] Chapman, Paul B., et al. "Improved survival with vemurafenib in melanoma with BRAF V600E mutation." New England Journal of Medicine 364.26 (2011): 2507-2516.

[6] Hauschild, Axel, et al. "Dabrafenib in BRAF-mutated metastatic melanoma: a multicentre, open-label, phase 3 randomised controlled trial." The Lancet 380.9839 (2012): 358-365.

[7] Young, Richard J., et al. "Loss of CDKN2A expression is a frequent event in primary invasive melanoma and correlates with sensitivity to the CDK4/6 inhibitor PD0332991 in melanoma cell lines." Pigment cell & melanoma research 27.4 (2014): 590-600.

[8] Dickson, Mark A. "Molecular pathways: CDK4 inhibitors for cancer therapy." Clinical Cancer Research 20.13 (2014): 3379-3383.

[9] Phase II clinical trial: NCT02202200: “Phase I-II Study With Tumor Molecular Pharmacodynamic (MPD) Evaluation and Pharmacokinetics of PD-0332991 in Patients Suffering Metastatic Melanoma (OPTIMUM)”

[10] Phase II clinical trial: NCT01037790: “Phase II Trial of the Cyclin-Dependent Kinase Inhibitor PD 0332991 in Patients With Cancer”

[11] Gozgit et al., “Ponatinib (AP24534), a multitargeted pan-FGFR inhibitor with activity in multiple FGFR-amplified or mutated cancer models.”, Mol Cancer Ther., 11(3):690-9 (2012).

[12] Clinical trial, phase I/II (recruiting): NCT02308553 – Clinical Phase I/II Study Investigating the Efficacy and Safety of Nintedanib Combined With Paclitaxel Chemotherapy for Patients With BRAF wt Metastatic Melanoma (NIPAWILMA)

[13] Clinical trial, phase I (recruiting): NCT02607813 – Phase I Study of LXH254 in Patients With Advanced Solid Tumors Haboring MAPK Pathway Alterations

[14] Clinical trial, phase I (recruiting): NCT02529553 – Clinical Phase I Study Investigating LY3076226 in Participants With Advanced or Metastatic Cancer

[15] Clinical trial, phase II (ongoing): NCT02316197 – Clinical Phase I Study Investigating MSC2490484A, an Inhibitor of a DNA-dependent Protein Kinase, in Advanced Solid Tumors or Chronic Lymphocytic Leukemia

[16] Kotula et al., “DNA-PKcs plays role in cancer metastasis through regulation of secreted proteins involved in migration and invasion.”, Cell Cycle., 14(12):1961-7 (2015)

[17] Clinical trial, phase I (recruiting): NCT02711345 – A Phase I Clinical Study With Investigational Compound LTT462 in Adult Patients With Specific Advanced Cancers

[18] Snyder et al., “Genetic basis for clinical response to CTLA-4 blockade in melanoma.”, New Engl J Med, 371(23):2189-2199 (2014).

[19] National Cancer Institute: GDC Data Portal (2017). TCGA SKCM. [online] Available at: https://portal.gdc.cancer.gov/projects/TCGA-SKCM [Accessed 2017-12-18].

[20] Li et al., “Standards and Guidelines for the Interpretation and Reporting of Sequence Variants in Cancer: A Joint Consensus Recommendation of the Association for Molecular Pathology, American Society of Clinical Oncology, and College of American Pathologists”, J Mol Diagn., 19(1):4-23 (2017).

[21] Dienstmann, R., et al. "Genomic aberrations in the FGFR pathway: opportunities for targeted therapies in solid tumors." Annals of oncology 25.3 (2013): 552-563.

[22] Ho, Han Kiat, et al. "Current strategies for inhibiting FGFR activities in clinical applications: opportunities, challenges and toxicological considerations." Drug discovery today 19.1 (2014): 51-62.

[23] Infante, Jeffrey R., et al. "A phase I study of the cyclin-dependent kinase 4/6 inhibitor ribociclib (LEE011) in patients with advanced solid tumors and lymphomas." Clinical Cancer Research (2016): clincanres-1248.

[24] Ascierto, Paolo Antonio, et al. "A phase Ib/II dose-escalation study evaluating triple combination therapy with a BRAF (encorafenib), MEK (binimetinib), and CDK 4/6 (ribociclib) inhibitor in patients (Pts) with BRAF V600-mutant solid tumors and melanoma." (2017): 9518-9518.

[25] Carlino, Matteo S., et al. "Differential activity of MEK and ERK inhibitors in BRAF inhibitor resistant melanoma." Molecular oncology 8.3 (2014): 544-554.

[26] Yadav, Vipin, et al. "Reactivation of mitogen-activated protein kinase (MAPK) pathway by FGF receptor 3 (FGFR3)/Ras mediates resistance to vemurafenib in human B-RAF V600E mutant melanoma." Journal of Biological chemistry 287.33 (2012): 28087-28098.
